# Covert cases of Severe Acute Respiratory Syndrome Coronavirus 2: An obscure but present danger in regions endemic for Dengue and Chikungunya viruses

**DOI:** 10.1371/journal.pone.0244937

**Published:** 2021-01-06

**Authors:** Lorenzzo Lyrio Stringari, Michel Norbim de Souza, Nésio Fernandes de Medeiros Junior, Jaqueline Pegoretti Goulart, Camila Giuberti, Reynaldo Dietze, Rodrigo Ribeiro-Rodrigues

**Affiliations:** 1 Laboratório Central de Saúde Pública do Estado do Espírito Santo, Secretaria de Estado da Saúde do Espírito Santo, Vitória, Brazil; 2 Núcleo de Doenças Infecciosas, Universidade Federal do Espírito Santo, Vitória, Brazil; 3 Secretaria de Estado da Saúde do Espírito Santo, Vitória, Brazil; 4 Instituto de Higiene e Medicina Tropical, Universidade Nova de Lisboa, Lisboa, Portugal; National Institute for Infectious Diseases Lazzaro Spallanzani-IRCCS, ITALY

## Abstract

**Background:**

The impact of SARS-CoV-2 in regions endemic for both Dengue and Chikungunya is still not fully understood. Considering that symptoms/clinical features displayed during Dengue, Chikungunya and SARS-CoV-2 acute infections are similar, undiagnosed cases of SARS-CoV-2 in co-endemic areas may be more prevalent than expected. This study was conducted to assess the prevalence of covert cases of SARS-CoV-2 among samples from patients with clinical symptoms compatible with either Dengue or Chikungunya viral infection in the state of Espírito Santo, Brazil.

**Methods:**

Presence of immunoglobulin G (IgG) antibody specific to SARS-CoV-2 nucleoprotein was detected using a chemiluminescent microparticle immunoassay in samples from 7,370 patients, without previous history of COVID-19 diagnosis, suspected of having either Dengue (n = 1,700) or Chikungunya (n = 7,349) from December 1^st^, 2019 to June 30^th^, 2020.

**Findings:**

Covert cases of SARS-CoV-2 were detected in 210 (2.85%) out of the 7,370 serum samples tested. The earliest undiagnosed missed case of COVID-19 dated back to a sample collected on December 18, 2019, also positive for Dengue Virus. Cross-reactivity with either Dengue virus or other common coronaviruses were not observed.

**Interpretation:**

Our findings demonstrate that concomitant Dengue or Chikungunya outbreaks may difficult the diagnosis of SARS-CoV-2 infections. To our knowledge, this is the first study to demonstrate, with a robust sample size (n = 7,370) and using highly specific and sensitive chemiluminescent microparticle immunoassay method, that covert SARS-CoV-2 infections are more frequent than previously expected in Dengue and Chikungunya hyperendemic regions. Moreover, our results suggest that SAR-CoV-2 cases were occurring prior to February, 2020, and that these undiagnosed missed cases may have contributed to the fast expansion of SARS-CoV-2 outbreak in Brazil. Data presented here demonstrate that in arboviral endemic regions, SARS-CoV-2 infection must be always considered, regardless of the existence of a previous positive diagnosis for Dengue or Chikungunya.

## Introduction

In December 2019, after several cases of severe pneumonia of unknown etiology emerged in China, the causative agent was y identified as SARS-CoV-2 (Severe Acute Respiratory Syndrome Coronavirus 2), a member of Coronaviridae family (Betacoronavirus genus) and associated with the onset of a severe respiratory disease termed “COVID-19” (COronaVIrus Disease 2019) [1–3]. In a matter of weeks, the SARS-CoV-2 outbreak spread rapidly, reaching nearly every country in the World, and compelling the World Health Organization (WHO) to declare COVID-19 a global pandemic by March 11^th^, 2020 [1, 4]. As of December 15, 2020, a cumulative total of nearly 71.3 million cases and 1,612,372 deaths have been reported since the start of the outbreak [5]. In Brazil, the first confirmed case of SARS-CoV-2 infection was announced by the Brazilian Ministry of Health on February 26, 2020; currently occupying the third place worldwide in number of cases, contributing with over 6.9 million cases and close to 181,835 deaths [6].

Symptoms associated with SARS-CoV-2 infection vary widely, from asymptomatic disease to multisystem organ failure and, even death [7]. However, in most cases, patients have few or no symptoms posing a challenge to prevent disease dissemination. The fast expansion of COVID-19 raised several public health concerns, one of them is associated with the possibility of misdiagnosing SARS-CoV-2 infections in regions where arboviral diseases, such as Dengue fever or Chikungunya, are concomitantly endemic [8]. In Brazil, endemic arboviral diseases have led to seasonal large scale outbreaks resulting in high rates of morbidity and mortality in the past two decades. Emergence of SARS-CoV-2 in arboviral–endemic areas has raised concerns regarding coinfection with the two viruses and the occurrence of misdiagnosis.

From November 1^st^, 2019 to March, 2020, while the first cases of SARS-CoV-2 were emerging across the globe, positive cases of Dengue and Chikungunya were soaring throughout Brazil. In the state of Espírito Santo it was not different. At the same time, 18.73% and 67.9% of individuals suspected of having either acute Dengue or Chikungunya infection, respectively, were positive for SARS-CoV-2 IgG. Parallelly, the first official known COVID-19 case in the state of Espírito Santo was reported on March 4^th^, 2020. A current threat in several countries, the impact on public health of concomitant Dengue, Chikungunya and COVID-19 outbreaks is still not fully understood. It has been reported that acute cases initially diagnosed as Dengue were later confirmed to be caused by SARS-CoV-2 [9–11]. Considering that the similarity of symptoms/clinical features shared by Dengue, Chikungunya and SARS-CoV-2 infections, it is fair to assume that misdiagnosis in co-endemic areas may be more prevalent than expected, especially when the majority of diagnosed cases are mainly based on clinical-epidemiological observations. Therefore, failing to consider SARS-CoV-2, as the etiological agent, due to the existence of a positive Dengue or Chikungunya test result may have serious consequences for both the patient and public health.

In the present work, we investigated the hypothesis that early covert SARS-CoV-2 cases were missed and that its diagnosis may have been hindered by an existing clinical or laboratorial diagnosis of acute Dengue or Chikungunya infections, in regions endemic for arboviral diseases. In order to confirm our hypothesis, serum samples from patients suspected of having Dengue or Chikungunya were re-tested for the presence of anti-SARS-CoV-2 antibodies, including the assessment of antibody cross-reactivity which might hinder diagnosis of COVID-19.

## Methods

### Study subjects

Serum samples from 7,370 patients with clinical symptoms compatible with either Dengue fever or Chikungunya were collected between December 1^st^, 2019 and June 30^th^, 2020 and tested for the presence of anti-SARS-CoV-2-nucleoprotein IgG antibodies. Inclusion criteria were: 1) samples from subjects with clinical symptoms compatible with Dengue and/or Chikungunya infection, 2) residents of the state of Espírito Santo, and 3) subjects with no prior history of COVID-19 diagnosis. Samples were anonymized prior to their inclusion in the study. SARS-CoV-2 IgG immunoreactivities were analyzed at the Espírito Santo’s Central Public Health Laboratory (LACEN-ES). Demographic data and laboratory results, including sample results (S), cutoff values (CO), and result Index values (S/CO) were obtained prior anonymization from laboratory information management database (Gerenciador de Ambiente Laboratorial–GAL, DATASUS, Ministry of Health).

### Ethics statement

LACEN-ES belongs to public health laboratories network coordinated by Brazilian Ministry of Health and is part of the state of Espírito Santo’s Secretary of Health. All samples used in the present work were stored at -80°C and after routine diagnosis for either Dengue or Chikungunya at the Immunology Division of Laboratório Central do Espírito Santo (LACEN-ES). The availability of these samples for research purpose during outbreaks is supported by Resolution number 510/2016 of the National Ethical Committee for Research–Brazilian Ministry of Health (CONEP–Comissão Nacional de Ética em Pesquisa, Ministério da Saúde, Brasil): which authorizes the use of clinical samples collected in the Brazilian Central Public Health Laboratories to accelerate the understanding, further the knowledge, contribute to public health surveillance and fast responses during outbreaks, epidemics or pandemic, such as the one observed for COVID-19.

### Detection of Chikungunya- and Dengue-specific IgM antibodies by ELISA

All samples used in the present work were previously tested for the presence of both Chikungunya and Dengue specific IgM antibodies by means of enzyme-linked immunosorbent assay (ELISA). Chikungunya diagnosis was carried out using an anti-Chikungunya virus ELISA (IgM) Test (cat #. EI 293a-9601 M, Euroimmun, Luebeck, Germany). ELISA sensitivity and specificity, according to the manufacturer, are 98.1% and 99.9%, respectively. For the Dengue diagnosis, samples were assayed using the anti-Dengue virus ELIS (IgM) Test (cat no. EI 266b-9601-1 M, Euroimmun, Luebeck, Germany). This test contains a microplate coated with viral particles and recombinant glycoprotein E (DEN 1–4). According to the manufacturer, sensitivity, and specificity, is 100% and 98%, respectively. Results interpretation for both assays were identical: values ≤ 0.8 were considered negative; between 0.8 and 1.1 borderline/inconclusive, and those ≥ 1.1 positive. Samples with borderline/inconclusive results were retested and the second result analyzed.

### Detection of SARS-CoV-2 IgG antibodies by chemiluminescent microparticle immunoassay

Presence of IgG specific to SARS-CoV-2 nucleocapsid protein N was detected by a highly specific and sensitive chemiluminescent microparticle immunoassay (CMIA, Abbott Laboratories SARS-CoV-2 IgG immunoassay, Abbott Park, IL). The Abbott Laboratories SARS-CoV-2 IgG assay is a two-step qualitative CMIA which employs acridinium ester-mediated chemiluminescence and is performed on the Architect i2000SR automated immunoassay analyzer. Testing was performed in accordance with the manufacturer’s instructions. The cutoff index (S/CO) is 1.4 and was calculated by dividing sample result (S) by the stored calibrator result (CO). Index ≥1.4 are considered positive, and Index < 1.4 are negative. Positive Percent Agreement (PPA) was 86.5% (95%CI: 65.09–97.09) for samples collected between 8–13 days post-symptom onset and 100% (95%CI: 95.89–100%) for those collected ≥14 days. Negative Percent Agreement (NPA) was 99.63% (95%CI: 99.05–99.90%). Results from 16 samples positive for IgG to SARS-CoV-2 collected between December 2019 and February, 2020 were confirmed using MAGLUMI™ 2019-nCoV IgG and IgM kits on a MAGLUMI™ 800 (Snibe, China), specific for both SARS-CoV-2 N and S proteins (sensitivity 80%, 95% CI:63–90; specificity: 99%, 95% CI: 92–100).

### Pre-SARS-CoV-2 pandemic Dengue positive serum samples

A pre-SARS-CoV-2-pandemic serum panel was used to assess Dengue and SARS-CoV-2 cross-reactivity. These samples were obtained from symptomatic, Dengue PCR positive patients, collected between 2009 and 2010 and stored at -80°C at the Núcleo de Doenças Infecciosas, Universidade Federal do Espírito Santo, UFES, Vitória, BRAZIL. A total of 84 serum samples (acute and convalescent paired sera) from 42 individuals (average age ± SD = 35.5 ±12 years, range 16–64 years; 16 males, and 26 females) was available. Dengue fever was confirmed by the detection of the viral genome by PCR and by multiple serology testing IgM and IgG ELISA (Table 1).

**Table 1:** Characteristics of matched, acute, and convalescent Dengue positive serum samples collected prior to the onset of the SARS-CoV-2-pandemic.

Sample ID	Gender	Age (Years)	Post-symptom onset (days)		qPCR Results		Dengue Serology Results						SARS-CoV-2 IgG Index (S/CO)		
							*Acute phase Samples*			Convalescent phase Samples					
			Acute Samples	Convalescent Samples	Result	Serotype	*Ag NS1*	*IgM*	*IgG*	Ag NS1	IgM	IgG	Acute	Convalescent	Results for both samples
**PPS-01**	Male	34	1	14	Pos	Dengue 2	*Pos*	*Neg*	*Neg*	Neg	Pos	Pos	0.34	0.29	Neg
**PPS-02**	Male	43	3	14	Pos	Dengue 2	*Neg*	*Neg*	*Neg*	Neg	Pos	Pos	0.12	0.21	Neg
**PPS-03**	Male	23	5	16	Pos	Dengue 2	*Neg*	*Neg*	*Neg*	Neg	Pos	Neg	0.13	0.12	Neg
**PPS-04**	Male	34	2	15	Pos	Dengue 2	*Pos*	*Neg*	*Neg*	Neg	Pos	Pos	0.16	0.08	Neg
**PPS-05**	Female	55	4	15	Pos	Dengue 2	*Neg*	*Neg*	*Neg*	Neg	Pos	Pos	0.14	0.18	Neg
**PPS-06**	Male	47	3	14	Pos	Dengue 2	*Neg*	*Neg*	*Neg*	Neg	Pos	Pos	0.21	0.05	Neg
**PPS-07**	Female	20	2	15	Pos	Dengue 2	*Neg*	*Neg*	*Neg*	Neg	Pos	Pos	0.09	0.08	Neg
**PPS-08**	Female	37	4	15	Pos	Dengue 1	*Pos*	*Neg*	*Neg*	Pos	Pos	Pos	0.17	0.04	Neg
**PPS-09**	Male	60	5	16	Pos	Dengue 2	*Pos*	*Neg*	*Pos*	Neg	Pos	Pos	0.15	0.04	Neg
**PPS-10**	Female	57	3	17	Pos	Dengue 2	*Neg*	*Neg*	*Neg*	Neg	Pos	Pos	0.19	0.2	Neg
**PPS-11**	Female	30	3	15	Pos	Dengue 2	*Neg*	*Neg*	*Neg*	Neg	Pos	Pos	0.13	0.1	Neg
**PPS-12**	Female	45	3	15	Pos	Dengue 2	*Neg*	*Neg*	*Neg*	Neg	Pos	Pos	0.07	0.1	Neg
**PPS-13**	Male	44	6	16	Pos	Dengue 2	*Pos*	*Neg*	*Pos*	Neg	Pos	Pos	0.14	0.19	Neg
**PPS-14**	Female	31	5	16	Pos	Dengue 2	*Neg*	*Pos*	*Pos*	Neg	Pos	Pos	0.95	0.76	Neg
**PPS-15**	Female	46	5	16	Pos	Dengue 2	*Neg*	*Pos*	*Pos*	Neg	Pos	Pos	0.27	0.3	Neg
**PPS-16**	Female	40	2	13	Pos	Dengue 2	*Neg*	*Neg*	*Neg*	Neg	Pos	Pos	0.09	0.08	Neg
**PPS-17**	Female	17	4	13	Pos	Dengue 2	*Neg*	*Neg*	*Neg*	Neg	Pos	Neg	0.05	0.16	Neg
**PPS-18**	Male	30	5	15	Pos	Dengue 2	*Neg*	*Pos*	*Pos*	Neg	Pos	Pos	0.14	0.21	Neg
**PPS-19**	Female	29	2	12	Pos	Dengue 2	*Neg*	*Neg*	*Neg*	Neg	Pos	Pos	0.1	0.09	Neg
**PPS-20**	Female	23	3	13	Pos	Dengue 2	*Neg*	*Pos*	*Pos*	Neg	Pos	Pos	0.1	0.12	Neg
**PPS-21**	Female	32	7	14	Pos	Dengue 2	*Neg*	*Pos*	*Pos*	Neg	Pos	Pos	0.12	0.02	Neg
**PPS-22**	Female	31	4	11	Pos	Dengue 2	*Neg*	*Pos*	*Pos*	Neg	Pos	Pos	0.14	0.13	Neg
**PPS-23**	Female	26	6	13	Pos	Dengue 3	*Neg*	*Pos*	*Pos*	Neg	Pos	Pos	0.43	0.21	Neg
**PPS-24**	Male	38	5	12	Pos	Dengue 2	*Neg*	*Neg*	*Neg*	Neg	Pos	Pos	0.15	0.05	Neg
**PPS-25**	Male	32	3	14	Pos	Dengue 2	*Neg*	*Neg*	*Neg*	Neg	Pos	Pos	0.08	0.02	Neg
**PPS-26**	Female	40	2	10	Pos	Dengue 2	*Neg*	*Neg*	*Neg*	Neg	Pos	Pos	0.15	0.03	Neg
**PPS-27**	Female	38	6	12	Pos	Dengue 2	*Neg*	*Pos*	*Pos*	Neg	Pos	Pos	0.2	0.06	Neg
**PPS-28**	Female	17	1	11	Pos	Dengue 2	*Neg*	*Neg*	*Neg*	Neg	Pos	Pos	0.29	0.15	Neg
**PPS-29**	Male	34	6	10	Pos	Dengue 3	*Neg*	*Pos*	*Pos*	Neg	Pos	Pos	0.13	0.03	Neg
**PPS-30**	Female	50	4	11	Pos	Dengue 1	*Pos*	*Neg*	*Neg*	Neg	Pos	Pos	0.18	0.07	Neg
**PPS-31**	Female	37	5	12	Pos	Dengue 2	*Neg*	*Pos*	*Pos*	Neg	Pos	Pos	0.15	0.1	Neg
**PPS-32**	Male	25	3	11	Pos	Dengue 2	*Pos*	*Neg*	*Neg*	Neg	Pos	Pos	0.11	0.08	Neg
**PPS-33**	Female	17	6	13	Pos	Dengue 2	*Neg*	*Neg*	*Pos*	Neg	Pos	Pos	0.25	0.23	Neg
**PPS-34**	Male	44	5	12	Pos	Dengue 2	*Neg*	*Neg*	*Neg*	Neg	Pos	Pos	0.1	0.15	Neg
**PPS-35**	Male	16	3	11	Pos	Dengue 2	*Neg*	*Neg*	*Pos*	Neg	Pos	Pos	0.1	0.1	Neg
**PPS-36**	Female	45	4	11	Pos	Dengue 2	*Neg*	*Pos*	*Pos*	Neg	Pos	Pos	0.11	0.24	Neg
**PPS-37**	Female	34	4	10	Pos	Dengue 2	*Neg*	*Neg*	*Pos*	Neg	Pos	Pos	0.21	0.2	Neg
**PPS-38**	Female	29	4	17	Pos	Dengue 2	*Pos*.	*Neg*	*Neg*	Neg	Pos	Pos	0.16	0.22	Neg
**PPS-39**	Male	48	3	16	Pos	Dengue 2	*Neg*	*Neg*	*Neg*	Neg	Pos	Pos	0.13	0.05	Neg
**PPS-40**	Female	64	3	15	Pos	Dengue 1	*Neg*	*Neg*	*Pos*	Neg	Pos	Pos	0.04	0.04	Neg
**PPS-41**	Female	27	7	12	Pos	Dengue 2	***NP***	*Neg*	*Neg*	***NP***	Pos	***NP ***	0.02	0.02	Neg
**PPS-42**	Male	23	4	18	Pos	Dengue 2	*Pos*	***NP***	***NP***	***NP***	***NP***	***NP***	0.02	0.02	Neg

### Statistical analyses

Statistical analyses were performed using GraphPad PRISM version 8.01 (GraphPad Software). ANOVA’s and Student’s t test were also performed. Categorical variables were compared using Fisher’s exact test and continuous variables with the Mann-Whitney U test. A *p* value ≤ 0.05 was judged statistically significant. Spearman’s correlation (rho value) was used to assess the relation between age and immunoreactivity Index (S/CO) and time post-symptom onset and immunoreactivity Index (S/CO).

### Role of the funding source

The funders facilitated data acquisition but had no role in the design, analysis, interpretation, or writing. The first two authors and the senior author (RR-R) had full access to all the data. The first four authors and the senior author (RR-R) have final responsibility for the decision to submit for publication.

## Results

**Fig 1:**
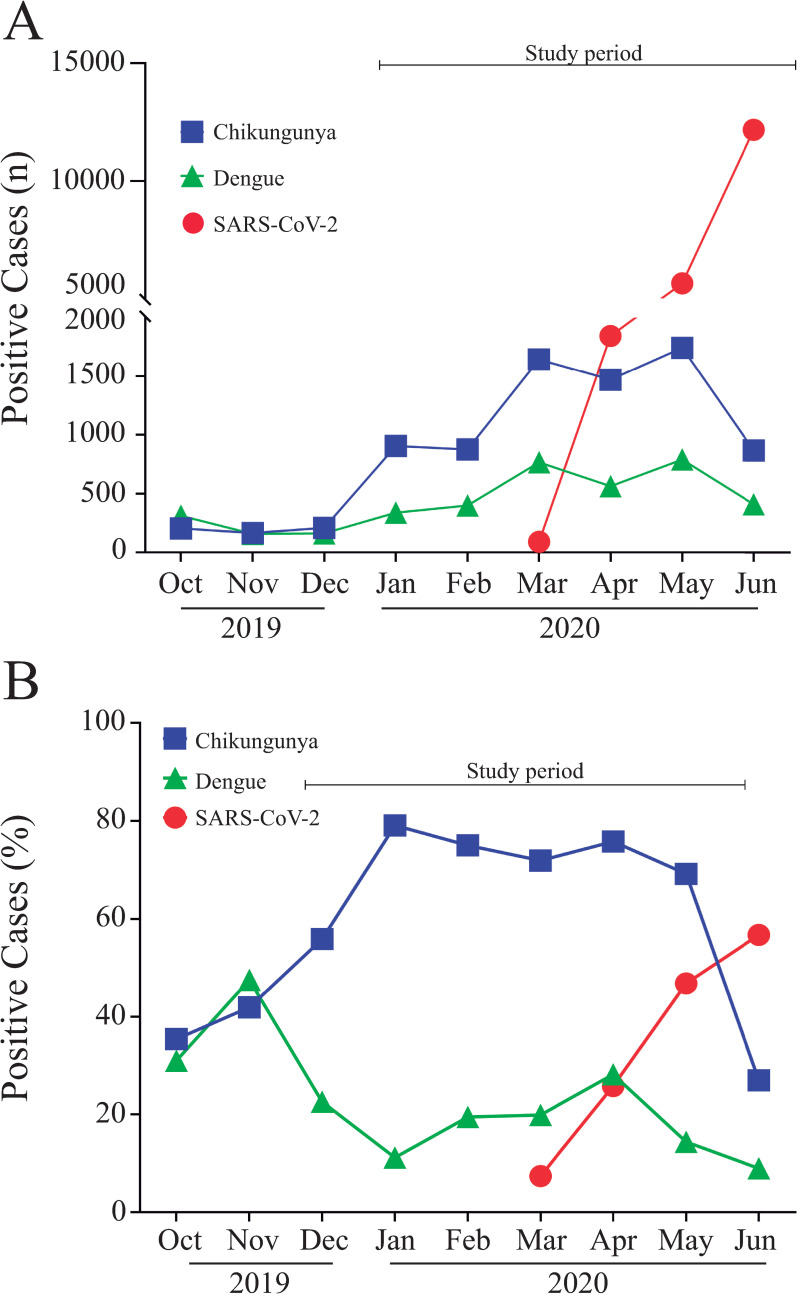
Characteristics of the Dengue, Chikungunya and SARS-CoV-2 outbreaks in the state of Espírito Santo from October 2019 to June 2020. A) Number of Dengue, Chikungunya and SARS-CoV-2 positive cases in Espírito Santo over time; B) Positivity rate of Dengue, Chikungunya and SARS-CoV-2 cases over time.

Considering that SARS-CoV-2 diagnosis in arboviruses hyperendemic regions could be challenging, prevalence of covert cases of SARS-CoV-2 was investigated in patients with acute symptoms compatible with either Dengue or Chikungunya infection, regardless of their previous arboviral serology result (n = 7,370). General characteristics of the studied population are given on Table 2. Serum samples from patients presenting symptoms compatible with either Dengue fever (n = 1,700) or Chikungunya (n = 7,349) infections, originally tested for the presence of specific IgM antibodies, were included in the present study (Fig 1A). IgM positivity rate in Chikungunya on samples collected from December, 2019 to June, 2020 was sustained high (69–79%), whereas among Dengue samples it was lower (11 to 28%) (Fig 1B). When the first SARS-CoV-2 case was officially announced by the Brazilian Ministry of Health on February 26^th^, 2020, incidence of Dengue and Chikungunya infections were soaring in the state of Espírito Santo. Soon after, on March 4^th^, the first case was reported in the state of Espírito Santo, skyrocketing to 19,761 cases by June 30 (Fig 1A); and from March to June 2020, SARS-CoV-2 positivity rate jumped from 7.4 to 56.7% (Fig 1B). No significant difference regarding age or gender distribution were observed when Dengue and Chikungunya groups were compared. Conversely, antibody positivity rate in Dengue and Chikungunya groups diverged; while presence of anti-Dengue IgM was confirmed on 228 (13.4%%) out of 1,700 samples tested, Chikungunya specific IgM antibodies were detected on 5,244 (74.4%) out of 7,349 samples (Table 2).

**Table 2:** General characteristics of the studied population.

Groups		Tested for Chikungunya		Tested for Dengue		Total Patients*	
**n (%)**		7,349 (99.72%)		1,700 (23.07%)		7,370 (100%)	
**Number of samples positive for arboviral disease (%)**		5,244 (71.35%)		228 (13.41%)		5,472 (74.25%)	
**Age (years) Average ± SD**		45.7 ± 17.25		41.3 ± 18.99		45.8 ± 17.26	
**Gender distribution n (%)**		**Female**	**Male**	**Female**	**Male**	**Female**	**Male**
		4,967 (67.6%)	2,382 (32.4%)	1,089 (67.5%)	600 (35.9%)	4,977 (67.5%)	2,393 (32.5%)
**Age/group (years) Average ± SD**		46.2 ± 16.5	44.8 ± 18.7	57.6 ± 16.7	48.4 ± 19.0	46.2 ± 16.5	44.8 ± 18.7
**Serology Results**	**Reactive Samples n (%)**	3,625 (72.34%)	1,619 (67.66%)	139 (12.76%)	89 (14.57%)	3,626* (72.8%)	1,622* (67.78%)
	**Non-reactive Samples n (%)**	1,282 (25.76%)	731 (30.55%)	873 (80.17%)	479 (78.4%)	1,290* (25.9%)	739* (30.88%)
	**Inconclusive Samples n (%)**	60 (1.21%)	32 (1.5%)	77 (7.07%)	43 (7.04%)	61* (1.23%)	32* (1.34%)

*—Serology results for the Total Patients’ group included samples from patients with clinical symptoms compatible with Chikungunya and/or Dengue.

**Fig 2:**
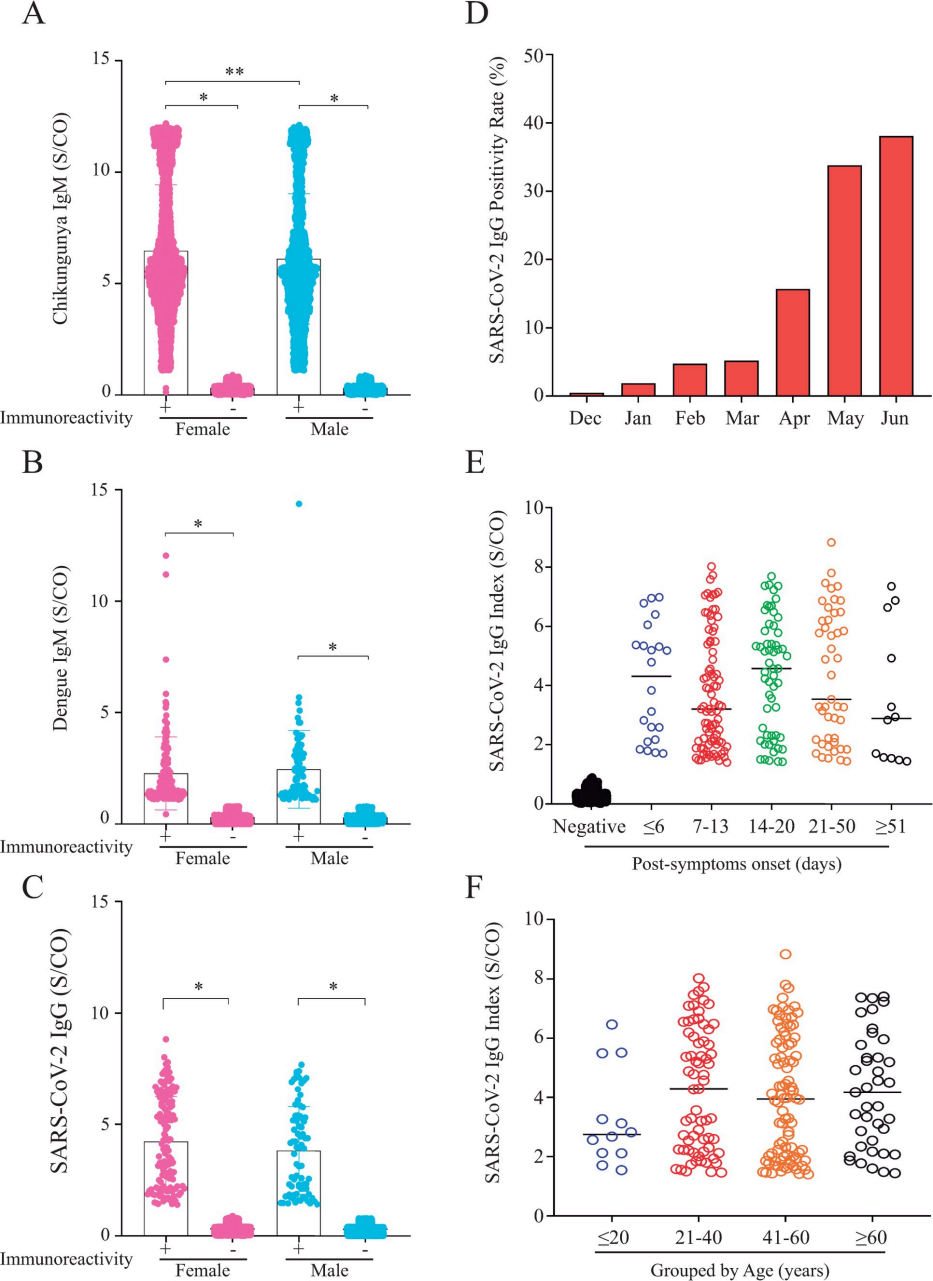
**A-C)** Assessment of gender influence on immunoreactivity levels in Chikungunya, Dengue, and SARS-CoV-2 patients. **A)** Chikungunya patients; **B)** Dengue patients; and **C)** SARS-CoV-2 patients. Categorical variables were compared using Fisher’s exact test and continuous variables with the Mann-Whitney U test; *p<0.05; **p<0.01. **2D**) Evolution of SARS-CoV-2 positivity rate from December 2019 to June 2020; **2E and F**) Assessment of SARS-CoV-2 IgG immunoreactivity index (S/CO) categorized by: **E)** days post-symptoms onset, and **F)** grouped by age.

**Fig 3:**
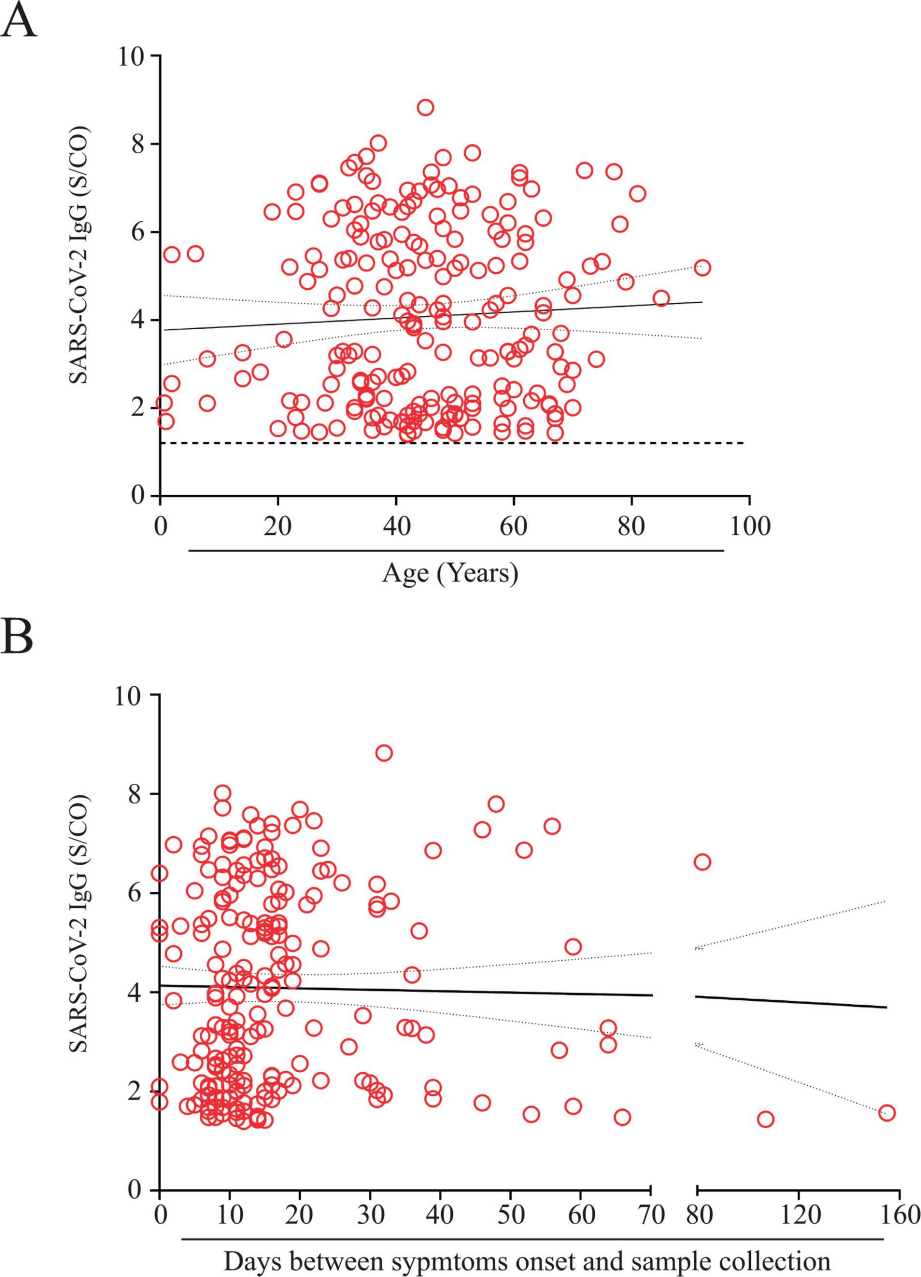
Correlation between SARS-CoV-2 IgG immunoreactivity index (S/CO), and both: **A)** age distribution (years). The black dotted line represents the cutoff point for the CMIA (S/CO = 1.4). **B)** time between symptoms onset and sample collection. No significant differences were observed. Rho (ᴩ) = 0.019 (Fig 3A) and 0.1111 (Fig 3B).

**Fig 4:**
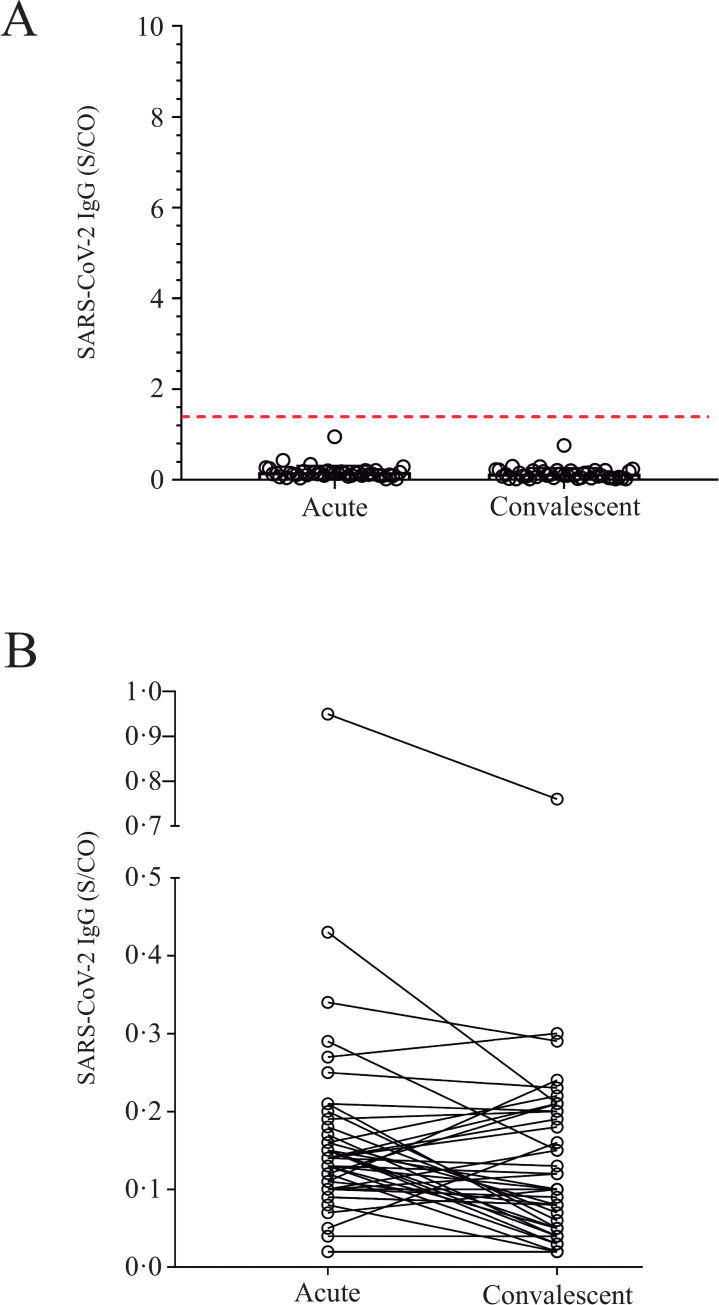
Assessment of pre-pandemic Dengue positive samples (PPS): **A)** Pre-pandemic samples (n = 84 samples) were tested using Abbott’s Architect SARS-CoV-2 IgG CMIA, 42 samples were from the acute phase and another 42 paired samples from the convalescent phase. The red dotted line represents the cutoff point for the CMIA (S/CO = 1.4); **B)** SARS-CoV-2 IgG immunoreactivity index (S/CO) displayed by acute samples and convalescent samples from Dengue positive patients. No significant differences were observed.

As depicted in Table 3, when samples were tested for the presence of anti-SARS-CoV-2 IgG antibodies 210 (2.85%) samples were positive; 133 (63.3%) were obtained from female patients aging 44.4 ± 17.3 years (average ± SD), while 77 (36.7%) were males aging 46.0 ± 14.8 years (average ± SD). Even though, female patients who tested for Chikungunya presented IgM immunoreactivity index values higher than those observed for males (p = 0.0067), gender did not affect either Dengue or SARS-CoV-2 IgG immunoreactivity indexes (Fig 2A–2C). Positivity rate for anti-SARS-CoV-2 IgG antibodies in the studied samples increased significantly from December 2019 (0.49%) to June 2020 (38.1%) (Fig 2D), nonetheless differences were not observed when SARS-CoV-2 IgG immunoreactivity index values were categorized by days-post-symptom onset or by age (Fig 2E and 2F). Additionally, no correlation between SARS-CoV-2 IgG immunoreactivity index and either days-post-symptom onset or age was found (Fig 3A and 3B). To exclude the possibility that the observed SARS-CoV-2 positive results were due to cross-reactivity with Dengue antibodies, as reported elsewhere [10, 12], a panel of pre-pandemic samples originated from a 2009−2010 Dengue outbreak in the state of Espírito Santo, including paired acute and convalescent phase sera from 42 Dengue fever patients (Table 1), was evaluated by CMIA. All 84 samples were negative for anti-SARS-CoV-2 IgG (Fig 4A and 4B), excluding the possibility of result misinterpretation caused by false-positive reactions.

**Table 3:** Characteristics of the SARS-CoV-2 IgG positive population.

Studied population	Tested for Chikungunya (n = 7,349)		Tested for Dengue (n = 1,700)		Total Patients (n = 7,370)	
Presence of anti-SARS-CoV2 IgG* **(%)**	176 (2.39%)		85 (5.00%)		210 (2.85%)	
Patients positive for SARS-CoV2 and arboviruses** **(%)**	68 (38.63%)		11 (12.94%)		79 (37.62%)	
**Immunoreactivity/Gender**	**Female**	**Male**	**Female**	**Male**	**Female**	**Male**
**Gender (%)**	49 (72.1%)	19 (27.9%)	7 (63.6%)	4 (36.4%)	133 (63.3%)	77 (36.7%)
**Age (years) Average ± S.D.**	43.7 ± 14.1	50.5 ± 13.2	45.6 ± 12.6	38.3 ± 12.6	44.4 ± 17.3	46.0 ± 14.8
**Anti-SARS-CoV2 IgG Positive Titer (Average ± SD)**	5.0 ± 3.5	5.4 ± 3.3	4.8 ± 1.9	2.9 ± 2.0	4.2 ± 2.0	3.8 ± 1.8
**Dengue or Chikungunya Specific IgM titer (DO/CO) Average ± SD**	6.5 ± 3.0	6.1 ± 2.9	2.1 ± 1.9	2.1 ± 1.1	N.A.	N.A.

*—Compared to the number of either Chikungunya (n = 7,349), Dengue. (n = 1,700) or Total samples tested (n = 7,370)

**—Compared to the total number of SARS-CoV2 IgG positive samples Chikungunya (n = 176), Dengue. (n = 85) or Total groups (n = 210).

N.A.–Not applicable.

Antibodies to SARS-CoV-2 16 were detected on samples collected before the first COVID-19 case was confirmed in Brazil, February 27, 2020 (Table 4). Furthermore, the earliest SARS-CoV-2 positive case, found in the studied population, dates back to December, 2019 and is represented by a sample by a 60-year-old female patient from Vitória exhibiting symptoms compatible with Dengue fever on December 9^th^ 2019, who had a blood sample taken on December 18^th^, testing positive for the presence of Dengue-specific IgM (S/CO = 4.31). However, when the same sample was evaluated for the presence of anti-SARS-CoV-2 IgG it was also positive (IgG S/CO = 2.24) (Table 4). To exclude the possibility that results were due to antigenic cross-reactivity with common coronaviruses, these 16 positive samples were also screened using a different CLIA method (Snibe Diagnostic, China—Maglumi2000 Plus 2019-nCov IgM and IgG assays). Anti-SARS-CoV-2 IgG and IgM were detected in 7 (43.8%) and 1 (6.3%) out of the 16 samples tested, respectively (Table 4), confirming that patients were exposed to SARS-CoV-2 earlier than officially announced in Brazil.

**Table 4:** Characteristics the first 16 SARS-CoV-2 positive cases.

Case #	Age (years)	Gender	Location	Sample Collection	Chikungunya		Dengue		SARS-CoV-2					
									Abbott Laboratories (nucleoprotein N)		Snibe Diagnostic (RBD)			
					IgM level	S/CO	IgM level	S/CO	IgG level	S/CO	IgG level	AU/mL	IgM level	AU/mL
**1**	60	Female	Vitória	12/18/19	NP	NP	**+**	4.31	**+**	2.42	**-**	0.47	**-**	0.45
**2**	8	Female	Vila Velha	12/27/19	**+**	1.25	NP	NP	**+**	2.11	**-**	0.26	**-**	0.24
**3**	53	Female	Vitória	1/15/20	**-**	0.33	NP	NP	**+**	1.57	**+**	3.16	**-**	0.23
**4**	51	Female	Vila Velha	1/21/20	**+**	11.82	**+**	1.16	**+**	6.78	**+**	3.16	**+**	1.17
**5**	40	Male	Vitória	1/22/20	**+**	2.18	NP	NP	**+**	2.70	**+**	1.54	**-**	0.38
**6**	62	Female	Guarapari	1/23/20	**+**	2.11	NP	NP	**+**	3.43	**+**	4.66	**-**	0.47
**7**	61	Female	Vitória	2/6/20	**+**	11.93	NP	NP	**+**	3.34	**+**	1.81	**-**	0.1
**8**	62	Male	Vitória	2/7/20	**+**	1.21	NP	NP	**+**	1.48	**-**	0.83	**-**	0.67
**9**	63	Male	Vitória	2/13/20	**+**	4.94	NP	NP	**+**	3.68	**-**	0.1	**-**	0.44
**10**	35	Female	Serra	2/13/20	**?**	0.99	NP	NP	**+**	2.24	**-**	0.41	**-**	0.02
**11**	46	Female	C. de Itapemirim	2/14/20	**+**	11.73	NP	NP	**+**	2.02	**-**	0.19	**-**	0.5
**12**	50	Female	Vitória	2/14/20	**+**	10.5	NP	NP	**+**	2.13	**+**	2.58	**-**	0.39
**13**	0.7	Male	Guarapari	2/17/20	NP	NP	**-**	0.05	**+**	2.12	**-**	0.09	**-**	0.39
**14**	44	Female	Vitória	2/19/20	**?**	0.99	NP	NP	**+**	4.35	**+**	4.44	**-**	0.3
**15**	63	Male	Colatina	2/19/20	NP	NP	**-**	0.61	**+**	2.17	**-**	0.12	**-**	0.34
**16**	43	Female	C. de Itapemirim	2/3/20	NP	NP	**-**	**-**	**-**	0.16	**-**	0.08	**-**	0.29
				2/27/20	**+**	11.12	NP	NP	**+**	1.93	**-**	0.08	**-**	0.21

NP—Not performed.

**Fig 5:**
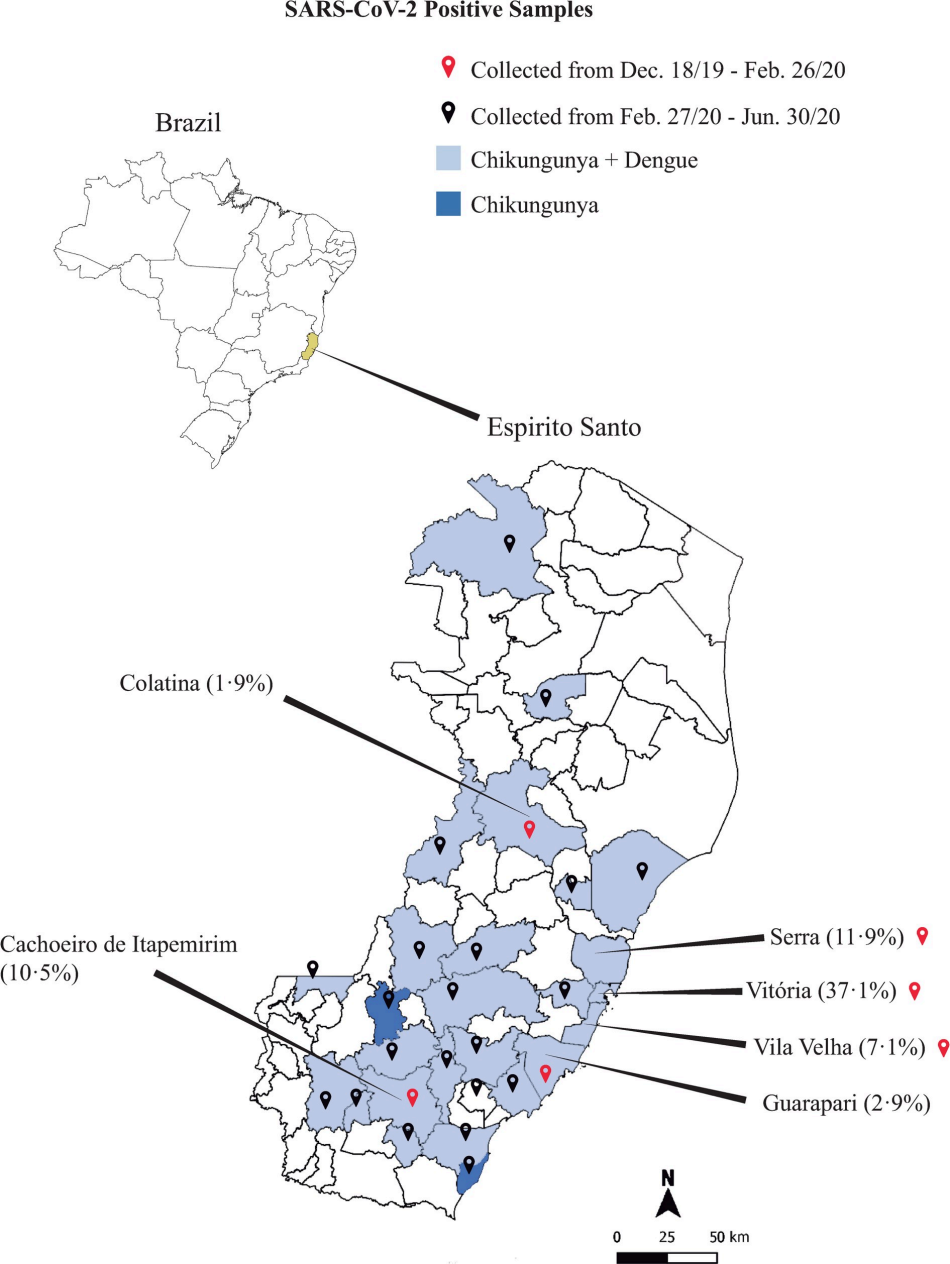
Distribution of the 210 positive cases of SARS-CoV-2 in the state of Espírito Santo. The red and black location markers represent the date of sample collection. The municipalities in dark blue represent the localities with cases of Chikungunya only, and municipalities in light blue where both Chikungunya and Dengue.

**Fig 6:**
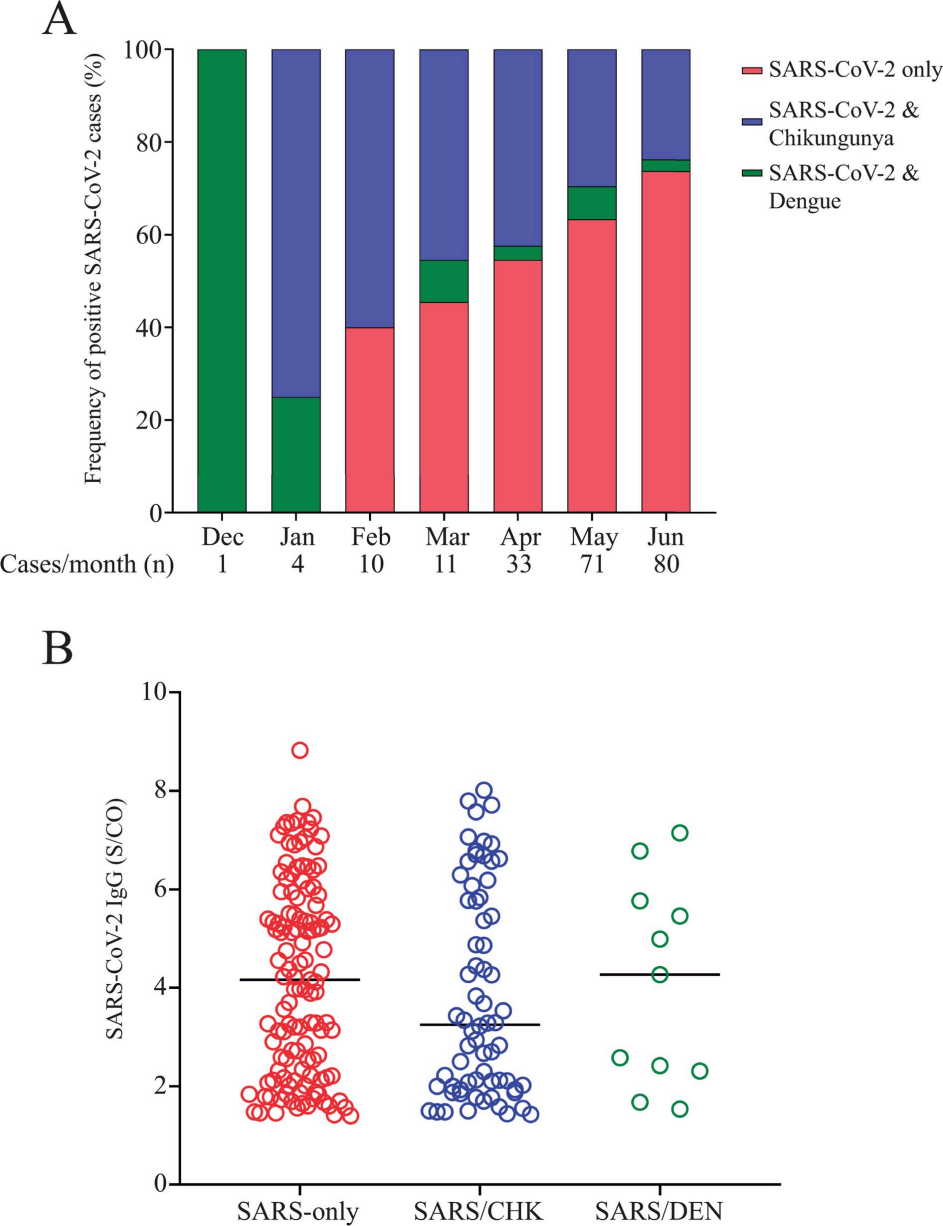
**A)** Frequency of positive SARS-CoV-2 cases over time (December, 2019 to June, 2020). SARS-CoV-2 cases were grouped in SARS-CoV-2-only (red bars), co-infected with Chikungunya (blue bars) or co-infected with Dengue virus (green bars). **B)** SARS-CoV-2 IgG immunoreactivity index (S/CO) was compared between groups SARS-CoV-2-only (red dots), and co-infected with Chikungunya (blue dots) or Dengue (green dots). No significant differences were observed.

**Fig 7:**
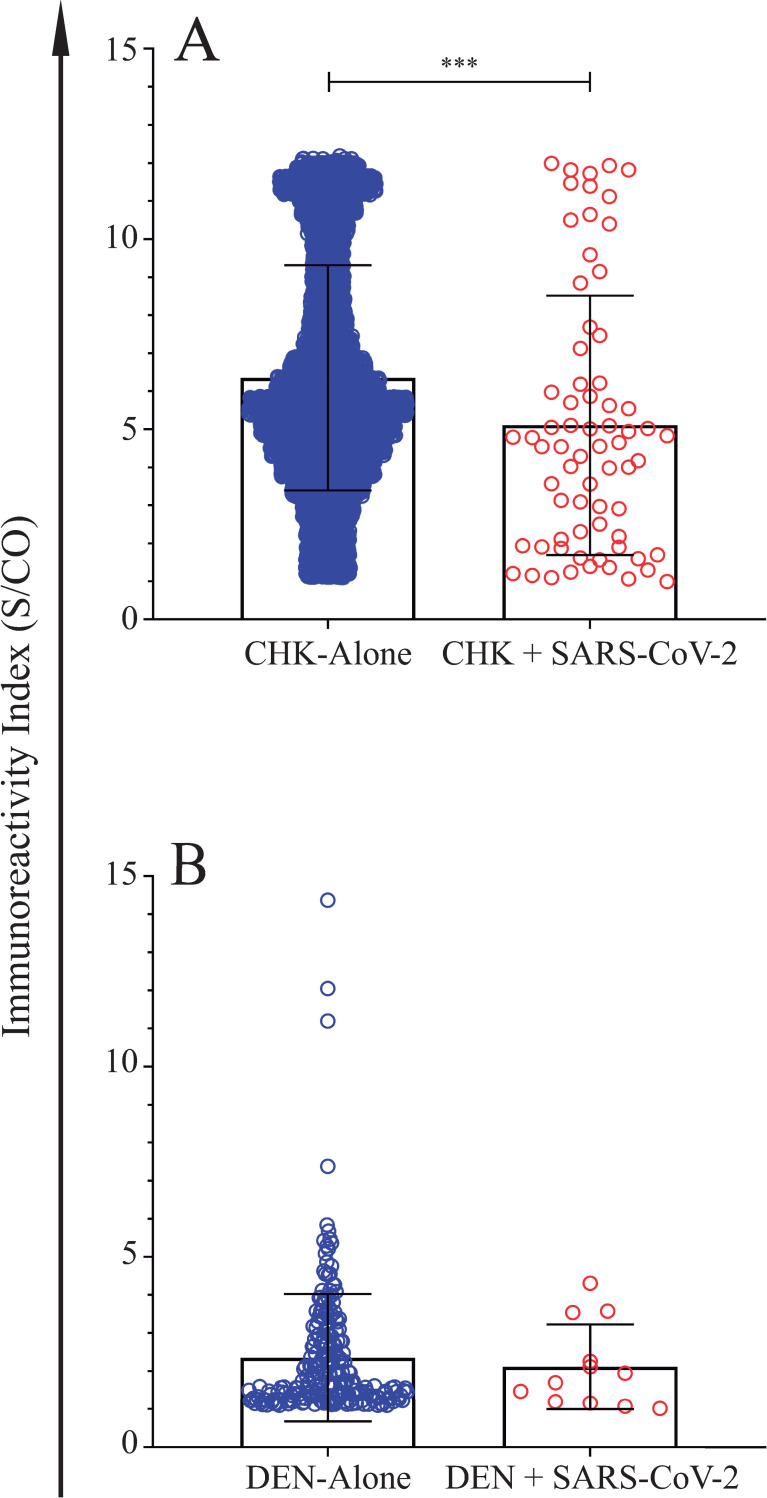
IgM immunoreactivity index in the presence or absence of SARS-CoV-2 IgG antibodies. **A)** Chikungunya positive cases. **B)** Dengue positive cases. Continuous variables were compared with the Mann-Whitney U test; ***p<0.0001.

In 2019, the population of the state of Espírito Santo state was estimated in 4,018,650 inhabitants. Our data showed that the highest number (n = 78, 37.1%) of SARS-CoV-2 positive cases were found in the capital city of Espírito Santo state, Vitoria, followed by the cities of Serra (n = 25, 11.9%), Cachoeiro do Itapemirim (n = 22, 10.5%), Vila Velha (n = 15, 7.1%), Viana (n = 13, 6.2%) and Guarapari (n = 6, 2.9%) (Fig 5). It is noteworthy to point out that from November, 2019 to June, 2020 the incidence of Dengue and Chikungunya was concomitant high in cities listed above. When Dengue, Chikungunya and SARS-CoV-2 data were compared, it became clear that co-positive cases, SARS-CoV-2/Dengue and SARS-CoV-2/Chikungunya, predominated from December, 2019 to January 2020 (Fig 6A). However, as the COVID-19 outbreak gained strength, frequency of SARS-CoV-2-only cases (patient positive for anti-SARS-CoV-2 IgG but negative for both Dengue IgM and Chikungunya IgM) increased from 0% to 73.8% (Fig 6A). Despite the increase in SARS-CoV-2-only cases, specific IgG immunoreactivity index were not affected by the absence or presence of IgM antibodies to both Dengue and Chikungunya, as depicted in Fig 6B. However, when IgM immunoreactivity indexes for both Dengue and Chikungunya were assessed either in the presence or absence of SARS-CoV-2 IgG antibodies, a significant reduction in IgM immunoreactivity index was observed for Chikungunya/SARS-CoV-2 samples (Fig 7A) but not for Dengue (Fig 7B).

## Discussion

Co-infection cases of SARS-CoV-2 and other intracellular pathogens have been reported elsewhere, ranging from arbovirus to protozoans [8, 13]. Considering that Malaria, Dengue, Chikungunya, and SARS-CoV-2 acute symptoms share similar clinical features, distinguishing them in co-endemic regions may pose a challenge with serious consequences [13, 14]. Data presented here demonstrate that 210 (2.85%) out of 7,730 patients, suspected of having either Dengue and Chikungunya infection, were positive for anti-SARS-CoV-2 IgG (Table 3), corroborating with the hypothesis that covert SARS-CoV-2 misdiagnosis is a present threat in Dengue and Chikungunya endemic regions [8]. Nevertheless, a high incidence (ranging from 40 to 73.8%) of covert SARS-CoV-2 cases was observed among patients initially suspected of having either Dengue fever or Chikungunya, but tested negative for both arboviruses. It is important to emphasize that 131 (62.4%) out of 210 SARS-CoV-2 positive patients displaying symptoms compatible with arboviral infection were negative for either Dengue or Chikungunya (Table 3), indicating that SARS-CoV-2 was not considered in the diagnostic strategy. Reported elsewhere [8, 10, 15], occurrence of serological cross-reactivity between Dengue and SARS-CoV-2 antibodies was not confirmed by our results (Table 1). Interestingly, 16 (7.6%) SARS-CoV-2 IgG positive samples were collected between December, 2019 and February, 2020, showing that exposure to SARS-CoV-2 was already occurring prior to COVID-19 was declared a global pandemic, and even before its presence was officially confirmed by the health authorities in Brazil. To rule out the possibility that those earlier SARS-CoV-2 cases were due to false-positive results, their samples were also screened for the presence of specific IgG to both N and S proteins by a different assay, and 7 (43.8%) out of the 16 samples tested positive (Table 4). Differences observed here, regarding Abbott’s CMIA and Snibe’s CLIA results, may be justified by the fact that anti-N IgG response is known to appear earlier than the response targeting the S protein [15].

Our data strongly supports the hypothesis that SARS-CoV-2 infection was overshadowed by a concomitant Dengue and Chikungunya epidemic. Case #16, depicted on Table 4, illustrates how an existent arboviral outbreak may hinder the diagnosis of SARS-CoV-2 infection. Briefly, a 43-year-old female patient, living in the city of Cachoeiro do Itapemirim, endemic for both Dengue and Chikungunya (Fig 5), presented clinical symptoms suggestive of Dengue fever on January 26, 2020. A blood sample (#1) was collected on February 3^rd.^, testing negative for anti-Dengue IgM. Symptoms persisted and another sample (#2) was collected on February 27^th^ and gave a positive result for anti-Chikungunya IgM (Table 4). When samples #1and #2 were later screened, presence of anti-SARS-CoV-2 IgG antibodies was detected on sample #2, suggesting that an acute SARS-CoV-2 infection was probably present and was missed when sample #1 was collected (Table 4). Such findings may help to explain why patients are diagnosed as Dengue fever at first and later confirmed to be COVID-19 cases [11, 16]. Therefore, presumption of Dengue or Chikungunya infection without further investigation may result in inadvertent omission of adequate standard of care for treatment and may ultimately facilitate the expansion of SARS-CoV-2 transmission 2 [8, 10].

It has been shown elsewhere that, after symptoms onset, antibody immunoreactivity indexes increase over time, with an enhanced clinical sensitivity of serological tests found ≥14 days after the beginning of clinical illness [15, 17, 18]. Analysis of anti-SARS-CoV-2 IgG S/CO data by days post-symptoms onset showed no significant difference, suggesting that all 210 IgG-positive patients were exposed ≥ 14 days before their blood samples were collected (Fig 2E). As a matter of fact, the earliest undiagnosed COVID-19 case dates back to December 18, 2019. Suspected of having Dengue Fever, our so called “patient zero”, tested positive for Dengue, and was only diagnosed positive for the presence of anti-SARS-CoV-2 IgG antibodies in the present study (Table 4). This finding raises concerns, particularly because clinicians frequently diagnose Dengue fever based only on the detection of IgM to Dengue virus, which may persist for months after resolution of viral infection [19].

In the present work, specific anti-SARS-CoV-2 IgG immunoreactivity was assessed by a highly specific and sensitive method (Abbott Laboratories SARS-CoV-2 IgG) CMIA [18, 20], thus the likelihood that positive results were triggered by serologic cross-reactivity with other coronaviruses or with arboviruses was negligible. According to different authors, cross-reactivity with other seasonal coronavirus, such as E229, OC43, HKU1, and NL63, was not observed when COVID-19 serum samples were tested by Abbott Laboratories SARS-CoV-2 IgG CMIA [21–23]. Occurrence of cross-reactivity between Dengue and SARS-CoV-2 antibodies [10, 12, 24], was not corroborated by results presented here as well as from other authors [25]. We demonstrated that all 84 (100%) Dengue-positive samples, collected between 2009 and 2010, tested negative for the presence of anti-SARS-Cov-2 IgG (Table 1; Fig 4). It is important to point out that previous reports suggesting cross-reactivity between Dengue and SARS-CoV-2 antibodies were based on limited sample size and exclusively on data acquired using lateral flow immunochromatographic rapid diagnostic test (RDT) [10, 12, 24]. In the early days of the COVID-19 pandemic, serology results were primarily obtained from RDT, which, despite a high specificity (≥99%), present a lower sensitivity when compared to CLIA/CMIA [25–27]; explaining why accuracy and reliability of RDT for Dengue and SARS-CoV-2 diagnosis are still so disputable [12, 24, 25, 28].

In addition to a high (2.85%) prevalence of patients with positive results for SARS-CoV-2 (Table 3), frequency of SARS-CoV-2-only cases (patients who are positive for anti-SARS-CoV-2 IgG but negative for both Dengue IgM and Chikungunya IgM) was also significantly elevated, comprising 73.8% of all samples tested in June 2020 (Fig 6A). Taken together these results corroborates with previous findings from the stringent “test-track-trace” strategy, which included large-scale PCR testing and extensive tracing technology, adopted by South Korean health authorities. As reported here, data from a study conducted in Daegu, South Korea, encountered a seroprevalence of 7.6%, with most of the IgG-positive individuals diagnosed as asymptomatic cases, indicating that the number of undiagnosed missing SARS-CoV-2 infections were higher than previously expected [29].

Although, proposed by Verduyn *et al* (2020) [30] that early high IgM and IgG levels observed in SARS-CoV-2 infection may increase disease severity in the case of Dengue co-infection, this hypothesis did not find support in our data. As shown here, IgM index on Chikungunya and SARS-CoV-2 co-infection samples was significantly reduced (p<0.0001) when compared to Chikungunya infection alone (Fig 7A), and undistinguishable when Dengue and Dengue/SARS-CoV-2 were compared (Fig 7B).

To our knowledge, this is the first study to investigate, with a robust sample size (n = 7,370) and using highly specific and sensitive CMIA method, the hypothesis that covert SARS-CoV-2 infections are more frequent than previously expected in Dengue and Chikungunya hyperendemic regions. Our findings demonstrated that an existing positive IgM test result for either Dengue or Chikungunya infections may prevent the early diagnosis of SARS-CoV-2 infections; since SARS-CoV-2 and arboviral diseases share similar features, the presence of a positive IgM result for either Dengue or Chikungunya may deceive clinicians to do not consider SARS-CoV-2 as a potential cause for the observed symptoms. Although, the main limitation of this study was the unavailability of paired nasopharyngeal swab samples, to confirm the presence of the virus, our results suggest that individual were exposed to SAR-CoV-2 prior to February, 2020, and that these covert cases may have contributed to the fast expansion of SARS-CoV-2 outbreak in Brazil.

In conclusion, our study reiterates the challenge associated with SARS-CoV-2 diagnose in endemic areas for arboviruses. Therefore, enforcement of vigilant awareness and systematic investigation is essential for appropriate treatment and control of SARS-CoV-2. Finally, data shown here raise the alert that in arboviral endemic regions, SARS-CoV-2 infection must be always considered, regardless of the existence of a previous positive test result for Dengue or Chikungunya and/or the absence of respiratory symptoms.
